# Review on Sol-Gel Synthesis of Perovskite and Oxide Nanomaterials

**DOI:** 10.3390/gels7040275

**Published:** 2021-12-18

**Authors:** Daniel Navas, Sandra Fuentes, Alejandro Castro-Alvarez, Emigdio Chavez-Angel

**Affiliations:** 1 Departamento de Química, Facultad de Ciencias Naturales, Matemática y del Medio Ambiente, Universidad Tecnológica Metropolitana, Las Palmeras 3360, Ñuñoa, Santiago 7800003, Chile; daniel.navas@utem.cl; 2 Departamento de Ciencias Farmaceúticas, Facultad de Ciencias, Universidad Católica del Norte, Av. Angamos 0610, Antofagasta 1270709, Chile; 3 Center for the Development of Nanoscience and Nanotechnology, CEDENNA, Av. Libertador Bernardo O’Higgins 3363, Santiago 9160000, Chile; 4 Laboratorio de Bioproductos Farmacéuticos y Cosméticos, Centro de Excelencia en Medicina Traslacional, Facultad de Medicina, Universidad de La Frontera, Av. Francisco Salazar 01145, Temuco 4780000, Chile; alejandro.castro.a@ufrontera.cl; 5 Catalan Institute of Nanoscience and Nanotechnology (ICN2), CSIC and BIST, Campus UAB, Bellaterra, 08193 Barcelona, Spain

**Keywords:** Sol-Gel, nanoparticles, perovskites, metal oxides

## Abstract

Sol-Gel is a low cost, well-established and flexible synthetic route to produce a wide range of micro- and nanostructures. Small variations in pH, temperature, precursors, time, pressure, atmosphere, among others, can lead to a wide family of compounds that share the same molecular structures. In this work, we present a general review of the synthesis of LaMnO_3_, SrTiO_3_, BaTiO_3_ perovskites and zinc vanadium oxides nanostructures based on Sol-Gel method. We discuss how small changes in the parameters of the synthesis can modify the morphology, shape, size, homogeneity, aggregation, among others, of the products. We also discuss the different precursors, solvents, working temperature, reaction times used throughout the synthesis. In the last section, we present novel uses of Sol-Gel with organic materials with emphasis on carbon-based compounds. All with a perspective to improve the method for future applications in different technological fields.

## 1. Introduction

**Figure 1:**
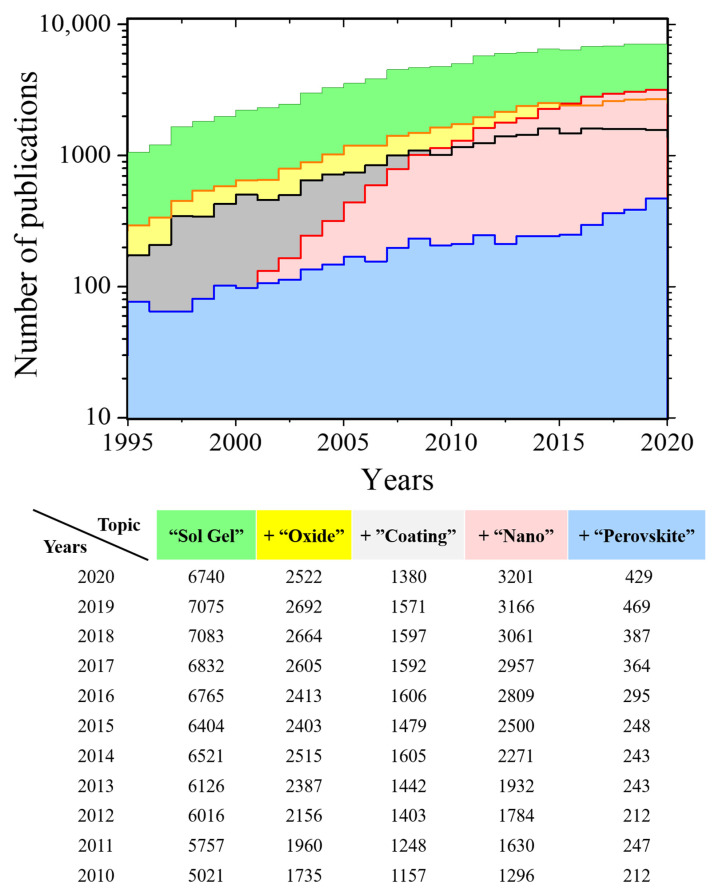
Top: Number of publications in the last 25 years including the keywords “Sol-Gel” (green), “Sol-Gel” + “oxide” (yellow), “Sol-Gel” + “coating” (grey), “Sol-Gel” + “nano” (red) and “Sol-Gel” + “perovskite” (blue). Bottom: table of number of publication in the with the number of publication in 2020–2010 period. All the data was extracted from web of science [17].

Sol-Gel method is one of the simplest techniques to synthesize high-quality nano and microstructures. This method provides several advantages over other synthesis routes such as control over the texture, size and surface properties of the materials, easy to implement, low cost, high quality, and production of materials with large surface areas [1]. This flexibility and simplicity make it very popular in the production of nanoscale powders [2,3,4] and its wide used as a coating method [5,6,7] (see Figure 1).

The first works on the Sol-Gel synthesis date from the mid-1800s with the pioneer studies of Ebelman [8] and Graham [9] on the fabrication of silica gels. The interest in gels grew very fast and notable researchers added their contributions to the field. Ostwald [10] and Lord Rayleigh [11] study the formation of Liesegang rings and the growth of crystals in gel potassium dichromate [12]. In the 1950s, Roy et al. extended its use and a large variety of ceramics were fabricated [13,14]. Years later, in the 1970s, Dislich presented new routes of synthesis of multicomponent oxide glasses [15] and the Sol-Gel method became a popular route to fabricate bulk glasses and ceramics. Since then, a number of researchers have contributed to the development of the Sol-Gel technology. A deep historical background of the Sol-Gel process and its evolution is presented by Sakka [16].

**Figure 2:**
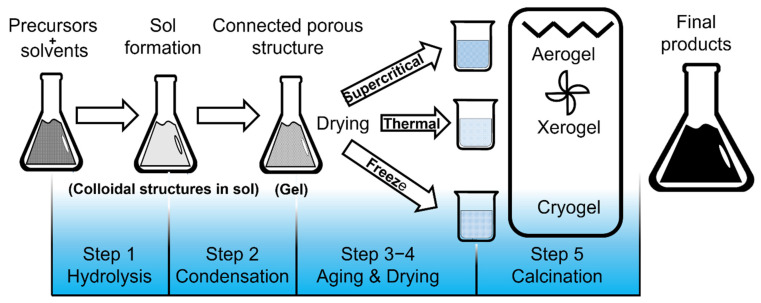
Schematic representation of step-by-step Sol-Gel method.

The Sol-Gel method involves the use of a colloidal solution (Sol) that evolves into a gel-like network including both a liquid and a solid phase as a result of several chemical reactions. It can be divided into two types: aqueous or hydrolytic and non-aqueous or non-hydrolytic. The aqueous Sol-Gel process can be described in five steps: hydrolysis, condensation, aging, drying and crystallization [18] (see Figure 2).

In the first step (hydrolysis) the metal alkoxide (MA) precursors and solvents are mixed. In this step, a nucleophilic attack takes place on MA by the oxygen from the water. As a result, simultaneous removal of alkoxide functional group and formation of alcohol functional group (M − OH) occurs. The hydrolysis and condensation reactions are strongly affected by process parameters such as the nature of the R-group, alkoxy functional group steric hindrance, temperature, pH, use of aqueous or non-aqueous media or organic solvents like alcohols, the ratio of solvent to MA or acetylacetonates as precursors and the use and concentration of catalysts (acid or a base). All of these variables can make synthetic control difficult through this route. The structure of the resulting gel is significantly different depending on the catalyst. This is due to the relative rates of the hydrolysis and condensation reactions. In general, the hydrolysis step gets progressively slower under acidic conditions and faster under basic conditions [19].

The condensation or polycondensation step is associated to the formation of a polymer network made of metal oxides linkages as a result of the elimination of solvent (water or alcohol) to form a sol. This process increases the viscosity of the solution as the polymer network grows, forming a porous structure within the liquid phase (Gel). The general chemical reaction for the hydrolysis and condensation process is given below:

$$M-\mathrm{OR}+H_{2}O\to \mathrm{MOH}+\mathrm{ROH}\;(\mathrm{hydrolysis})$$

MOH+XO-M→MOM+XOH (condensation)

where *M* = metal, *X* = *H* or alkyl Group (C*_n_*H_2*n*+1_). During the aging step, polycondensation continues within the localized solution along with precipitation of the gel network, which decreases porosity and increases thickness between colloidal particles. Another important factor is physical treatment of the sol or gel. Something as simple as evaporation rate during gelation can have a substantial impact on gel structure. When the liquid is removed from the gel, major changes to the network structure may occur. If the structure is maintained, an aerogel is formed—on the contrary if the structure collapses, a xerogel is formed. If the liquid is removed at low temperatures the term cryogels is coined. The aerogel shows high pore volume and surface area and xerogel results in low surface area and pore volume. Heat treatment is also important for drying gels as well as removing surface hydroxyl groups, densifying the material to produce a ceramic monolith or converting to a crystalline material [1].

Compared with other methods, the Sol-Gel method has many unique advantages, e.g.,:

1. Possibility of obtaining special products such as powders, films or coatings, microspheres, fibers;
2. Obtaining new solids with improved properties;
3. High purity and homogeneity of the materials obtained;
4. Saving energy during the process;
5. Full control over the particle size and morphology;
6. The solution and reaction step allows to incorporate easily, uniformly and quantitatively some trace elements, achieving a uniform doping at the molecular level.
7. Compared with the solid-phase reaction, the chemical reaction is simpler and only requires a lower synthesis temperature (<220 °C).

But also some disadvantages such as, e.g.,:

1. Very sensible to moisture;
2. Difficult to scale up;
3. Can include several steps and is a time-consuming process;
4. Dimension and volume changes during different steps.

## 2. Synthesis of Perovskites

Alkoxide, alkoxide-salt, and Pechini methods are the most popular Sol–Gel-based techniques used in the synthesis of perovskites. The Sol-Gel Pechini method (chelate poly esterification) became the most used due to its versatility in preparing perovskite membranes, depositing dielectric films for the production of capacitors and multicomponent oxide materials [20]. One of the most significant advantages of this process is its simplicity and the low-temperature precursor handling. This results in the fabrication of nanopowders with excellent purity and uniformity, as well as precise control of the final composition of the material. Pechini method is also a popular choice for the synthesis of various mixed oxides due to its capacity to combine chemicals (such as lactic, glycolic, citric, and EDTA acids) leading to the creation of polybasic acid chelates with dissolved cations [21]. Chelating agents are employed to prevent partial metal segregation in the final compound, which might occur as a result of various interactions between metal ions in the solution [22]. The polyesterification of chelates occurs when a polyhydroxy alcohol is added to the solution and heated, resulting in a cross-linked chain of metal atoms connected to organic radicals [14]. When two chelating agents work together to complex all metal ions, a precursor solution is formed with all metal ions entirely bound. As a result, a more stable chelate complex system develops and the polymerization is aided by the following addition of ethylene glycol, which enhances uniformity. This method tends to reduce metal segregation after decomposition under heat treatment settings. In the synthesis process and subsequent heat treatment, the ratio of metal precursors to chelating agents is critical. In theory, this ratio should be high enough to ensure that all metal ions are tightly bound to their structures and prevents precipitates in the solution.

The Sol-Gel Pechini method offers an excellent control over perovskite structure producing very homogeneous solutions. At temperatures near 1000 °C, this approach results in the creation of pure crystal perovskite structure.

Using this method, Shlapa et al. [23] synthesized very homogenous La_1-x_Sr_x_MnO_3_ (with 0.23 < *x* < 0.25) nanoparticles with a very narrow particle sizes distribution between 30–35 nm. They reported that the precursor arises during the reaction between metal ions and organic compounds (citric acid and ethylene glycol) and pyrolysis of the resulting gel. The crystalline perovskite structure emerged in one step at around 600 °C, and the level of crystallinity increased as the treatment temperature was increased.

Zhang et al. [24] studied a large variety of novel rare earth-doped (Dy^3+^, Eu^3+^, Sm^3+^, Tb^3+^, Yb^3+^/Er^3+^, Yb^3+^/Tm^3+^, and Yb^3+^/Ho^3+^) La_4_Ti_3_O_12_ perovskite-like structures. The as-synthesized phosphors exhibit characteristic down-conversion (DC) or up-conversion (UC) emissions corresponding to the rare earth activator ions upon UV or NIR excitation.

Lima et al. [25] investigated the preparation of red-emitting LaAlO_3_:Eu^3+^ and green-emitting LaAlO_3_:Tb^3+^ phosphors using a modified Pechini’s synthesis and sorbitol as polyalcohol chelating agents. The authors showed that the use of sorbitol as polyalcohol instead of ethylene glycol increases luminescence. A comparison of the Sol-Gel and Sol-Gel Pechini method is given in Table 1.

**Figure 3:**
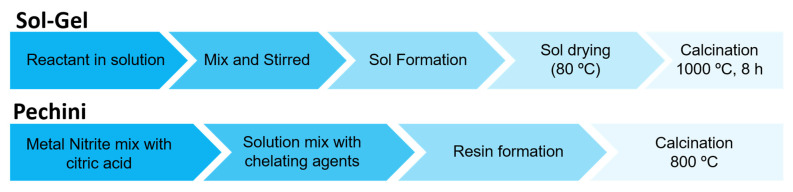
Summary of steps for each perovskite synthesis method discussed.

Recently, Osman et al. [26] reported the preparation of Y^3+^ doped BaCe_0.54_Zr_0.36_Y_0.10_O_2.95_ (BCZY) powders with various chelating agents as: citric acid, tartaric acid, glycolic acid, nitriloacetic acid, ethylenediaminetetraacetic acid and triethylenetetramine (TETA). The influence of the molecular weight of the chelating agent and functional group on thermal decomposition, phase formation and morphology of the samples were studied. The different types of chelating agents significantly affected the elemental composition, especially on B site of ABO_3_ perovskite structure of BCZY. The prepared powders using chelating agents from carboxylic acid and polyamino carboxylic groups formed Zr-cluster and Ce-cluster respectively, giving rise to the fluctuation in the calculated elemental mole fractions. The use of TETA in the synthesis minimized the formation of BaCO_3_, leading to the formation of high percentage of BCZY perovskite phase and homogeneous elemental composition. A summary of Pechini and conventional Sol-Gel method is shown in the Figure 3.

### 2.1. Sol-Gel Synthesis of Strontium and Barium Titanates and Its Derivate

Strontium and barium titanates (SrTiO_3_ and BaTiO_3_, respectively) are widely used in the industry and in medical areas. BaTiO_3_ is a low-cost ferroelectric material with a positive temperature coefficient, high dielectric constant and non-linear optical. It is also used as catalyst, microwave absorber and dielectric component of class II ceramic [27]. On the other hand, SrTiO_3_ is a well-known perovskite material for quantum paraelectricity with an unusually high dielectric constant [28]. SrTiO_3_ can be also found as a base material in high-voltage capacitors, voltage-dependent resistors and substrates for superconductors [29]. Despite the fact that SrTiO_3_ does not show ferroelectric properties at room temperature, it is possible to induce ferroelectric behavior by doping, substitution, among other factors. SrTiO_3_ and BaTiO_3_ and their derivates are very promising for gas sensing applications [30] and as n-type thermoelectric materials [31].

Size effects in nanostructured materials are of great importance from both fundamental considerations and practical applications. Initial research on size effects in ferroelectric materials has been mainly concentrated on BaTiO_3_ (BT). It is well-known that ferroelectricity properties strongly depend on its microstructure (crystallinity and grain size) and decreases with decreasing particle (grain) size, and disappears below a certain critical dimensions [32]. Then, the preparation methods play a critical role to define the desired properties. The traditional preparation methods of perovskite-type powders are performed by solid state reactions at high temperatures, usually higher than 1000 °C, and under specific pressure and pH conditions. This method involves several stages, such as the mixing of the raw material, calcinations at high temperatures, washing, milling, classification, filtering, drying and sifting. However, it is difficult to control the morphology and size of particles of these materials causing a diminution or destruction of the ferroelectric behavior [33,34].

Another approach is the synthesis in solution, where the titanates are prepared through coprecipitation and pyrolysis of metallo-organic precursors or through Sol-Gel processes. In general, the process uses titanium alkoxide and Ba (or Sr) salts, with and without occurrence of organic species. The reaction modifies and stabilize cations agents preventing the accumulation of the particles [35,36]. The main advantages of this method are: low working temperatures, large control of the particle size (from 10 to 100 nm) and the possibility of synthesize mixed oxide networks. However, starting products are expensive and the synthesis can be challenging.

The hydrothermal synthesis is another popular option, this method uses alkoxide or oxidizes as titanium source in presence of a salt from the alkaline-earth metal ion (halide, acetate, nitrate or hydroxide). The reaction occurs in alkaline conditions (pH = 13–14) in a temperature range from 90 °C to 200 °C. The main advantage of this method is that the experimental procedure is less complex and uses cheaper starting products than the Sol-Gel method. The main disadvantage of the hydrothermal method is that the titanates synthesized crystallize, mostly, in the metastable cubical phase which does not exhibit ferroelectric properties [37,38,39,40]. On the other hand, the use of water as reaction medium can also induce the incorporation of OH species to the BaTiO_3_ structure, which in turn leads to defects in the network [32,41]. These defects also modified the chemical stoichiometry and reduce the quality of the final products.

A popular way to produce these oxide titanates is by reaction of titanium *n*-butoxide (Ti(OC_4_H_9_)_4_), tetrachloride (TiCl_4_) or TiOSO_4_ with metal-nitrate M(NO_3_)_2_ [42], -chloride MCl_2_ [43] or -salts M(OH)_2_ [44] solutions (with M = Mg, Ca, Sr, Ba, Mn, etc.) through a Sol-Gel synthesis. In general oxides titanates are synthesized using Sol-Gel method to yield a crystalline material at much lower temperatures than usually required for solid-state reactions [45]. This method involves the use of hydrolysis to form gels from metal oxides before subjecting them to post processing to obtain high purity titanates.

Fuentes et al. [43] synthesized high-purity BaTiO_3_ (BT) using a Sol-Gel-hydrothermal reaction of TiCl_4_ and a BaCl_2_ solution in an oxygen atmosphere to obtain pseudo-tetragonal BT and particle sizes < 100 nm. The authors studied the effect of temperature (70–220 °C) and the incorporation of an oxygen partial pressure (60 bar) on the transition of BT from cubic to a pseudo-tetragonal phase. The results demonstrated that the oxygen partial pressure plays a crucial role in the BT nanoparticle formation under hydrothermal conditions, advantaging the increase of the ferroelectric exchange interactions, which are mediated by the oxygen ions, influencing of this mode, in the ferroelectric properties of BT. Using the same method, Fuentes et al. also synthesized SrTi_(1-x)_O_3_:Fe*_x_* powders with *x* = 0, 1, 3 and 5 mol% Fe, Ba_1−x_Sr*_x_*TiO_3_ (*x* = 0–1) and BaTiO_3_-ZnO:Eu heterostructures [46,47,48]. The results indicated increasing substitutional doping of Fe in SrTiO_3_ and that Sr ions were in the 2+ oxidation state in the perovskite structure, Fe participate as a mixture of Fe^3+^ and Fe^4+^ oxidation states, increasing the formation of oxygen vacancies as the Fe content increased in the sample. On the other hand, the reactant type used: BaCl_2_ and SrCl_2_ or Ba(OH)_2_ and Sr(OH)_2_ salts, directly influences the chemical and structural properties of Ba_1−x_Sr*_x_*TiO (BST) nanoparticles grown. The presence of Cl^−^ ions resulted in a better Sr incorporation into the network of BST when the Ba:Sr mole ratio used in the starting reactants was less than one and the presence of OH^−^ ions led to BST nanoparticles with less structural defects and oxygen vacancies.

### 2.2. Synthesis of LaMnO_3_ Nanoparticles

LaMnO_3_ (LMO) is a perovskite with the general formula ABO_3_. Each B atom occupies the vertex of the perovskite lattice and six oxygen atoms surround it to form an octahedron. The A element occupies the center of eight octahedrons. The oxidation state of B cation can be modulated by varying preparation steps during the perovskite synthesis, such as temperature [21], non-stoichiometry of cations A or B [12,14] or substituting La^3+^ by lower oxidation state cations, such as Ca^2+^, Ba^2+^ or Sr^2+^, or even higher oxidation state cation such as Ce^4+^. Sol-Gel technique is one of the easiest and most efficient methods to synthesize different LaMnO_3_ NPs. Several groups have reported the formation of LaMnO_3_ nanostructures via Sol-Gel method [49,50,51].

The typical studies focus on the optimization of the synthesis conditions during the Sol-Gel process to improve the oxidation capacity (Mn^3+^/Mn^4+^ mixed oxidation state), including complexant agent to metal nitrate ratio, pH of the gel and calcination protocol to get pure perovskites with no phase segregation and enhanced textural properties. Xue et al. [52] obtained nanocrystalline LMO powders by Sol-Gel combustion method. They reported the effect of citric acid (CA) to metal nitrates (MN) molar ratio of the precursor solution on the structure of gels and the powders. The crystallite size of the as-formed LMO powders increases with increasing the CA/MN molar ratio from 30 at 100 nm. In a similar study, Miao et al. [53] prepared modified LMO perovskites with porous tubular skeleton structure. They integrated this NPs into a double network hydrogel and dispersed in polyacrylamide (PAM)/polyvinyl alcohol (PVA) matrixes to prepare a flexible supercapacitor. The LMO-PAM/PVA hydrogel electrodes showed a significant improvement in mechanical properties and charge-discharge cycle stability. These encouraging results were attributed to the double network structure of the hydrogel electrodes and the introduction of LMO nanoparticles.

Liu et al. [54] produced LMO of high surface area using CA and EDTA as chelating agents. They found that EDTA-citrate produced smaller NPs than the single citrate acid. The samples exhibited a high specific surface area showing excellent oxygen reduction and evolution reaction catalytic performance.

In another work, Shaterian et al. [55] prepared LMO nanoparticles via Sol-Gel process using stearic acid as complexing reagent. The as-synthesized nanoparticles showed pure perovskite structure which presented a rhombohedral structure with average particle sizes of 20–30 nm. More recently, Onrubia-Calvo et al. [56] prepared a series of Sr-doped La_1−x_Sr_x_MnO_3_ perovskites (x = 0.1, 0.2, 0.3, 0.4 and 0.5) by varying citrate to nitrate molar ratio in the starting solution, pH and calcination protocol.

Conversely, Chaozhu Shu et al. [57] used a facile nonstoichiometric strategy to introduce A-site cationic vacancies in LaMnO_3_, La_0.9_MnO_3_, La_0.7_MnO_3_, perovskite oxides were synthesized by combining Sol-Gel method and thermal treatment process in air. This procedure provides the advantage of great specific surface area and abundant cationic La vacancies on the surface, La_0.7_MnO_3−__δ_ (L_0.7_MO) can provide effective active sites for oxygen electrode reactions and a large storage space for Li_2_O_2_ accommodation.

## 3. Synthesis of Vanadium Oxides

Vanadium oxides display a wide range of oxides states ranging from V^2+^ to V^5+^, leading to different valence and mix valence states. V_2_O_5_ and their variations V_2_O_5_·nH_2_O (xerogel), NH_4_VO_3_, VO(OR)_3_ and VOCl_3_ are well-known as intercalation host lattices. Many guest molecules can be reversibly intercalated in the oxide layers under soft chemical reactions [58]. Which, in turn, induces structural changes on the vanadium oxides and different shapes and morphologies can be obtained [59]. Under hydrothermal treatment, novel structures have been reported. These structures feature a mix oxidation state ratio (V^3+^ to V^5+^), however, the relation between morphology and oxidation state is still under debate.

For the Sol-Gel synthesis an alkoxide is used as precursor which, after hydrolysis and condensation reactions, can lead to different vanadium morphologies. Normally this process can be tuned with hydrothermal treatment varying the reaction times and the temperature. A simple route to obtain xerogel is described as follows [60]:

$$V_{2}O_{5\;\left(s\right)}+6\;{\left({\mathrm{CH}}_{3}\right)}_{3}{\mathrm{COH}}_{\left(l\right)}\overset{\;\mathrm{Reflux}\;}{\to }2\;\mathrm{VO}{\left(\mathrm{OC}{\left({\mathrm{CH}}_{3}\right)}_{3}\right)}_{3\;\left(l\right)}+3{\;H}_{2}O_{\left(l\right)}$$

By adding water, the hydrolysis reaction takes place

$$\mathrm{VO}{\left(\mathrm{OC}{\left({\mathrm{CH}}_{3}\right)}_{3}\right)}_{3\;\left(l\right)}+{\;H}_{2}O_{\left(l\right)}\overset{}{↔}\mathrm{VO}\left(\mathrm{OH}\right){\left(\mathrm{OC}{\left({\mathrm{CH}}_{3}\right)}_{3}\right)}_{2\;\left(l\right)}+{\left({\mathrm{CH}}_{3}\right)}_{3}{\mathrm{COH}}_{\left(l\right)}$$

The condensation reaction immediately runs forming the layered xerogel

$$\mathrm{VO}{\left(\mathrm{OC}{\left({\mathrm{CH}}_{3}\right)}_{3}\right)}_{3\;\left(l\right)}+\mathrm{VO}\left(\mathrm{OH}\right){\left(\mathrm{OC}{\left({\mathrm{CH}}_{3}\right)}_{3}\right)}_{2\;\left(l\right)}\overset{}{↔}\;{\left({\left({\mathrm{CH}}_{3}\right)}_{3}\mathrm{CO}\right)}_{3}\mathrm{OV}-O-\mathrm{VO}{\left(\mathrm{OC}{\left({\mathrm{CH}}_{3}\right)}_{3}\right)}_{2\;\left(l\right)}+{\left({\mathrm{CH}}_{3}\right)}_{3}{\mathrm{COH}}_{\left(l\right)}$$

$$\mathrm{VO}\left(\mathrm{OH}\right){\left(\mathrm{OC}{\left({\mathrm{CH}}_{3}\right)}_{3}\right)}_{2\;\left(l\right)}+\mathrm{VO}\left(\mathrm{OH}\right){\left(\mathrm{OC}{\left({\mathrm{CH}}_{3}\right)}_{3}\right)}_{2\;\left(l\right)}\overset{}{↔}{\left({\left({\mathrm{CH}}_{3}\right)}_{3}\mathrm{CO}\right)}_{2}\mathrm{OV}-O-\mathrm{VO}{\left(\mathrm{OC}{\left({\mathrm{CH}}_{3}\right)}_{3}\right)}_{2\;\left(l\right)}+{\;H}_{2}O_{\left(l\right)}$$

**Figure 4:**
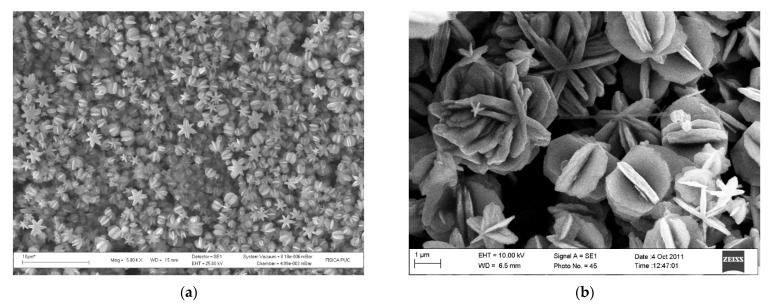
SEM Micrographs of Vanadium oxides: (**a**) High yield synthesis of vanadium oxides nanocogs. (**b**) V_6_O_11_ architecture rotationally six folds layered material.

Xerogel is used to develop many vanadium oxides nanostructures, alkylamines and thiols (usually 1-octadecanethiol) have been successfully intercalated between the layers. Once thiols formed the layered nanocomposite with xerogel, the compound is submitted to hydrothermal treatment at 180 °C during seven days. This process induces six folds symmetric cogs [61] and the star fruit morphology [62] (see Figure 4).

**Figure 5:**
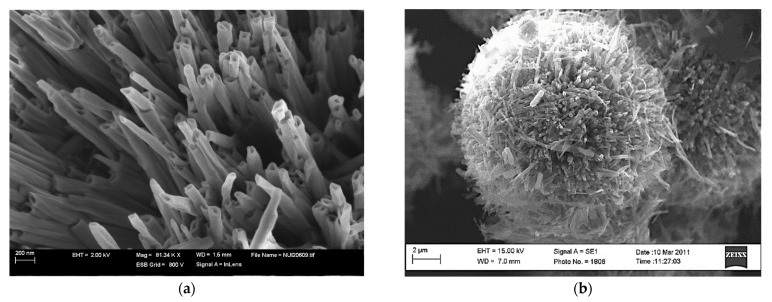
SEM Micrographs vanadium oxides: (**a**) nanotubes, (**b**) center hollowed vanadium oxide nanourchin.

Other morphologies that can be synthetized via Sol-Gel are nanotubes and nanourchins [63,64]. The first stage of the synthesis starts with the alkoxyde (VO(OCH(CH_3_)_2_)_3_) precursor in presence of alkylamine (e.g., hexadecylamine CH_3_(CH_2_)_14_CH_2_NH_2_)). The water and ethanol turns into an intercalated alkylamine–V_2_O_5_ layered nanocomposite. Subsequently, the reaction is subjected to hydrothermal treatment for seven days at 180 °C. The composites rolls up originating vanadium oxide nanotubes or nanourchins [65] (high density vanadium oxide nanotubes arrangement spherical clusters) which only differs on the stoichiometry molar proportion (see Figure 5).

Hydrolysis stage:

$$\mathrm{VO}{\left(\mathrm{OCH}{\left({\mathrm{CH}}_{3}\right)}_{2}\right)}_{3\;\left(l\right)}+{\;H}_{2}O_{\left(l\right)}\overset{}{↔}\;\mathrm{VO}\left(\mathrm{OH}\right){\left(\mathrm{OCH}{\left({\mathrm{CH}}_{3}\right)}_{2}\right)}_{2\;\left(l\right)}+{\left({\mathrm{CH}}_{3}\right)}_{2}{\mathrm{CHOH}}_{\left(\mathrm{ac}\right)}$$

Condensation stages

$$\mathrm{VO}{\left(\mathrm{OCH}{\left({\mathrm{CH}}_{3}\right)}_{2}\right)}_{3\;\left(l\right)}+\mathrm{VO}\left(\mathrm{OH}\right){\left(\mathrm{OCH}{\left({\mathrm{CH}}_{3}\right)}_{2}\right)}_{2\;\left(l\right)}\overset{}{↔}\;{\left({\left({\mathrm{CH}}_{3}\right)}_{2}\mathrm{HCO}\right)}_{2}\mathrm{OV}-O-\mathrm{VO}{\left(\mathrm{OCH}{\left({\mathrm{CH}}_{3}\right)}_{2}\right)}_{2\;\left(l\right)}+{\left({\mathrm{CH}}_{3}\right)}_{2}{\mathrm{CHOH}}_{\left(\mathrm{ac}\right)}$$

$$\mathrm{VO}\left(\mathrm{OH}\right){\left(\mathrm{OCH}{\left({\mathrm{CH}}_{3}\right)}_{2}\right)}_{2\;\left(l\right)}+\mathrm{VO}\left(\mathrm{OH}\right){\left(\mathrm{OCH}{\left({\mathrm{CH}}_{3}\right)}_{2}\right)}_{2\;\left(l\right)}\overset{}{↔}{\left({\left({\mathrm{CH}}_{3}\right)}_{2}\mathrm{HCO}\right)}_{2}\mathrm{OV}-O-\mathrm{VO}{\left(\mathrm{OCH}{\left({\mathrm{CH}}_{3}\right)}_{2}\right)}_{2\;\left(l\right)}+H_{2}O_{\left(l\right)}$$

**Figure 6:**
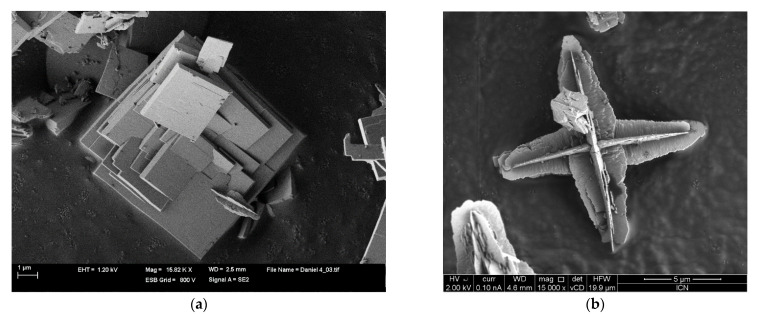
SEM Micrographs of: (**a**) (NH_4_)_2_V_7_O_16_ microsquares, and (**b**) Isolated VO_2_ cross.

Another interesting microstructure are (enH_2_V_7_O_16_) and (NH_4_)_2_V_7_O_16_ layered-compounds [66,67]. These compounds are synthesized using V_2_O_5_ precursor with ethylenediamine (en) suspended in a mixture of water-ethanol. Then the mixture is treated with acetic acid or NH_4_VO_3_ with NaBH_4_ in water. The synthesis is aided with hydrothermal treatment. Another procedure to obtain (NH_4_)_2_V_7_O_16_ was performed by Navas et al. [68], they treated the NH_4_VO_3_ precursor with acetic acid in presence of 1–hexadecylamine dissolved in ethanol. Then, the vanadium alkoxyde was obtained by decreasing the pH of the system. Once water is added, an intercalation compound is formed under the Sol-Gel process. A hydrothermal treatment for at least 24 h at 180 °C yields squared layered structures of different sizes (see Figure 6). This morphology can be modified into VO_2_ crosses by changing the reaction time and working temperature (10 days and 200 °C). The whole process can be described as follows:

$${\mathrm{NH}}_{4}{\mathrm{VO}}_{3\;\left(\mathrm{ac}\right)}+3{\mathrm{CH}}_{3}{\mathrm{COOH}}_{\left(\mathrm{ac}\right)}\overset{}{\to }\;\mathrm{VO}{\left({\mathrm{OOCCH}}_{3}\right)}_{3\;\left(\mathrm{ac}\right)}+{\;\mathrm{NH}}_{4}{\mathrm{OH}}_{\left(\mathrm{ac}\right)}+H_{2}O_{\left(l\right)}$$

Hydrolysis stage

$$\mathrm{VO}{\left({\mathrm{OOCCH}}_{3}\right)}_{3\;\left(\mathrm{ac}\right)}+H_{2}O_{\left(l\right)}\overset{}{↔}\mathrm{VO}\left(\mathrm{OH}\right){\left({\mathrm{OOCCH}}_{3}\right)}_{2\;\left(\mathrm{ac}\right)}+{\;\mathrm{CH}}_{3}{\mathrm{COOH}}_{\left(\mathrm{ac}\right)}$$

Condensation stages

$$\mathrm{VO}{\left({\mathrm{OOCCH}}_{3}\right)}_{3\;\left(\mathrm{ac}\right)}+\mathrm{VO}\left(\mathrm{OH}\right){\left({\mathrm{OOCCH}}_{3}\right)}_{2\;\left(\mathrm{ac}\right)}\overset{}{↔}{\left({\mathrm{OOCCH}}_{3}\right)}_{2}\mathrm{OV}-O-\mathrm{VO}{\left({\mathrm{OOCCH}}_{3}\right)}_{2\;\left(\mathrm{ac}\right)}+{\;\mathrm{CH}}_{3}{\mathrm{COOH}}_{\left(\mathrm{ac}\right)}\;$$

$$\mathrm{VO}\left(\mathrm{OH}\right){\left({\mathrm{OOCCH}}_{3}\right)}_{2\;\left(\mathrm{ac}\right)}+\mathrm{VO}\left(\mathrm{OH}\right){\left({\mathrm{OOCCH}}_{3}\right)}_{2\;\left(\mathrm{ac}\right)}\overset{}{↔}{\left({\mathrm{OOCCH}}_{3}\right)}_{2}\mathrm{OV}-O-\mathrm{VO}{\left({\mathrm{OOCCH}}_{3}\right)}_{2\;\left(\mathrm{ac}\right)}+H_{2}O_{\left(l\right)}$$

## 4. Synthesis of Zinc Oxides

Throughout the years, ZnO nanostructures and nanoparticles (ZnO NPs) have been a subject of research for promising applications in many technological, chemical and biological fields [69,70,71,72,73,74,75]. The different chemical routes to produce ZnO have been incorporated to produce 1D (nanotubes, nanoparticles, nanowires, nanorods) [3,76,77,78,79,80], 2D (nanosheets, nanoplates and nanopellets) and many 3D self-assemble with novel morphologies flowers, urchins, and dandelions [75,81,82,83]. Even though, these fascinating nanostructures can offer novel properties to ZnO, some defects are often found in the shape, size distribution and homogeneity. In general, these defects have a correlation with the chemical synthetic approach, starting from the Zn^2+^ salts precursors, pH, solvents, reaction time and temperature, additives like surfactants and other organic molecules, etc. [84,85,86]. One of the main challenges with these synthetic procedures is to control the size distributions, which can be vary from a few nanometers to micrometers. In terms of synthesis, the precursor Zn(CH_3_COO)_2_·2H_2_O has been widely used because it provides a moderate hydrolysis step, which can be controlled using organic molecules (e.g., surfactants or glycols), nitrogenated amines (used as the precipitating agent) or ethanol. This process originates ZnO NPs with a narrow size distribution ranging from 14 to 70 nm [87,88,89].

A short and simple Sol-Gel route to obtain ZnO nanostructures was reported by Yung–Kuan Tseng et al. [90] The authors described a hydrolysis and condensation processes from zinc acetate dihydrate with different types of glycols: ethylene glycol (EG), glycerol (GLY) and diethylene glycol (DEG) performed under reflux for one hour and increasing the temperature up to 160 °C (heating rate used 1 °C/minute). The final products were treated under two different heating processes, dripped onto silicon wafers at 160 °C and calcined at 500 °C. Under these conditions they generated three different morphologies with each solvent: fibers, flakes and spheres. The fabrication steps are given by:

Step 1

$$\mathrm{Zn}{\left({\mathrm{CH}}_{3}\mathrm{COO}\right)}_{2\;}+\mathrm{HO}-{\mathrm{CH}}_{2}{\mathrm{CH}}_{2}-\mathrm{OH}\overset{\;\mathrm{Reflux}\;}{\to }{\;\mathrm{Zn}}^{2+}\cdot {}^{-}{\mathrm{OCH}}_{2}{\mathrm{CH}}_{2}O^{-}+2{\mathrm{CH}}_{3}{\mathrm{COO}}^{-}+2H^{+}$$

Step 2

$$\frac{3}{2}\mathrm{Zn}{\left({\mathrm{CH}}_{3}\mathrm{COO}\right)}_{2\;}+\mathrm{HO}-{\mathrm{CH}}_{2}{\mathrm{CHOHCH}}_{2}-\mathrm{OH}\overset{\;\mathrm{Reflux}\;}{\to }\;\frac{3}{2}{\mathrm{Zn}}^{2+}\cdot {}^{-}{\mathrm{OCH}}_{2}{\mathrm{CHOHCH}}_{2}O^{-}+3{\mathrm{CH}}_{3}{\mathrm{COO}}^{-}+3H^{+}$$

$$\mathrm{Zn}{\left({\mathrm{CH}}_{3}\mathrm{COO}\right)}_{2\;}+\mathrm{HO}-{\mathrm{CH}}_{2}{\mathrm{CH}}_{2}{\mathrm{OCH}}_{2}{\mathrm{CH}}_{2}-\mathrm{OH}\overset{\;\mathrm{Reflux}\;}{\to }{\;\mathrm{Zn}}^{2+}\cdot {}^{-}{\mathrm{OCH}}_{2}{\mathrm{CH}}_{2}{\mathrm{OCH}}_{2}{\mathrm{CH}}_{2}O^{-}+2{\mathrm{CH}}_{3}{\mathrm{COO}}^{-}+2{\;H}^{+}$$

**Figure 7:**
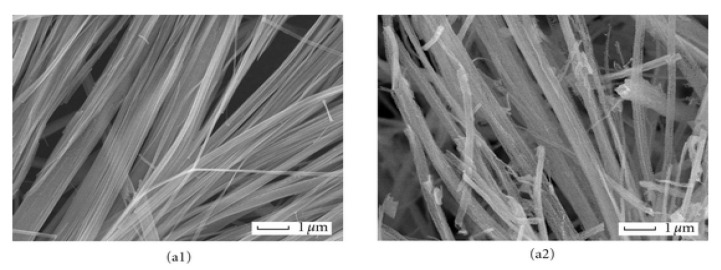

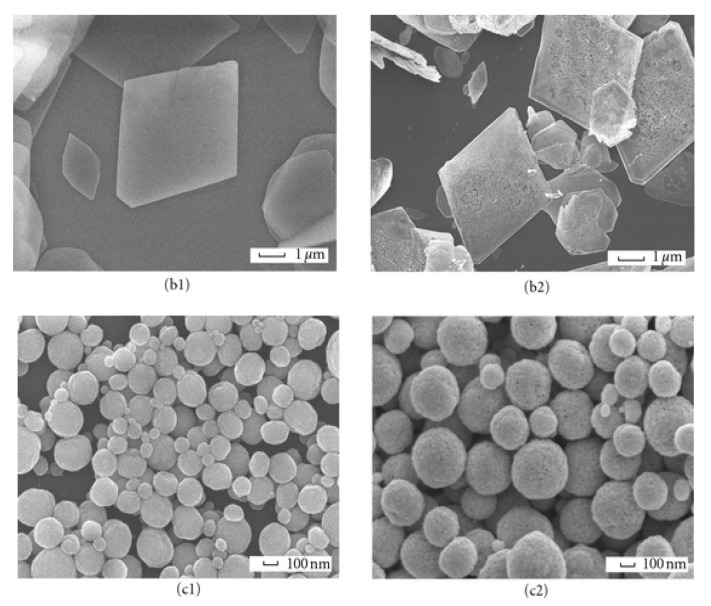
SEM images of different ZnO nanstructures obtained with different glycol solvents and heated at 160 °C (1) and calcined at 500 °C (2): (**a1**,**a2**) ethylene glycol, (**b1**,**b2**), glycerol and (**c1**,**c2**) diethylene glycol solvents. Adapted from [90].

The fibers are formed by the assembly of several unit structures of Zn^2+^ and the solvents (EG, GLY and DEG). For EG-based structures, Zn^2+^ −^−^OCH_2_CH_3_O^−^ terminal oxygen ions are formed, depending on temperature used at the final stage different shapes can be obtained. Samples dried at 160 °C reveal smooth surface, whereas the samples annealed at 500 °C in air results into small ZnO particles (see Figure 7a1,a2).

Another popular synthesis is the precipitation method using a titration to ensure a controlled Sol-Gel reaction. Md Jahidul Haque et al. [91] reported a synthesis route using Zn(CH_3_COO)_2_·2H_2_O as precursor mixed with NaOH dissolution at 35 °C. Once mixtures turn homogeneous, methanol was added dropwise under vigorous stirring for 90 min until a white gel was formed. The gel was dried overnight at 80 °C and calcinated at 250 °C during four hours. The reaction is given by:

$$\mathrm{Zn}{\left({\mathrm{CH}}_{3}\mathrm{COO}\right)}_{2}\times 2{\;H}_{2}O_{\left(\mathrm{aq}\right)}+2{\;\mathrm{NaOH}}_{\left(\mathrm{aq}\right)}\overset{{\mathrm{CH}}_{3}\mathrm{OH}}{\to }{\;\mathrm{ZnO}}_{\left(s\right)}+2{\;\mathrm{CH}}_{3}{\mathrm{COONa}}_{\left(\mathrm{aq}\right)}+H_{2}O_{\left(l\right)}$$

This synthesis was performed using a strong base which has the ability to ionize completely in water. Shokuhfar et al. [92], on the other hand, performed the Sol-Gel processes using a weak base which usually developed a partial ionization (%I < 5%). They used triethanolamine (N(CH_3_CH_2_OH)_3_, TEA) as surfactant, with methanolic solutions of Zn(CH_3_COO)_2_·2 H_2_O at different ratios. The resultant solution was kept at 50 °C all night allowing the completion of hydrolysis and condensation reactions. The final product was calcinated at 500 °C and ZnO Nps were obtained. The increase of the surfactant concentration led to a smaller particle size with narrow size distribution and enhanced homogeneity.

Azimul Haque and Mahalakshmi [93] performed the precipitation processes avoiding the Sol-Gel step. The authors used the same weak base with a partial ionization (<5%), TEA as a complexating agent (Lewis base role, electron pair donor) in combination with a strong base NaOH and Zn(CH_3_COO)_2_·2H_2_O as Zn^2+^ source. Zn^2+^ and TEA reagents were mixed and stirred at 80 °C bath. NaOH was added drop by drop until pH reaches 11. A white precipitate was washed up several times and dried at 60 °C. The TEA was used to obtain wurtzite structure. The precipitation was tuned by changing the temperature, pH and synthetic procedures.

For this method two mechanisms have been proposed:

Mechanism 1

$$\mathrm{Zn}{\left({\mathrm{CH}}_{3}\mathrm{COO}\right)}_{2}\times 2{\;H}_{2}O_{\left(\mathrm{aq}\right)}+2{\;\mathrm{TEA}}_{\left(\mathrm{aq}\right)}\overset{}{\to }\;\mathrm{Zn}{\left[\mathrm{TEA}\right]}_{\left(\mathrm{aq}\right)}+2{\;\mathrm{CH}}_{3}{\mathrm{COO}}_{\left(\mathrm{ac}\right)}^{-}$$

$$\mathrm{Zn}{\left[\mathrm{TEA}\right]}_{\left(\mathrm{aq}\right)}\overset{}{\to }{\;\mathrm{Zn}}_{\left(\mathrm{ac}\right)}^{2+}+{\mathrm{TEA}}_{\left(\mathrm{aq}\right)}$$

$$2{\;\mathrm{NaOH}}_{\left(s\right)}\overset{{\;H}_{2}O\;}{\to }\;2{\;\mathrm{Na}}_{\left(\mathrm{ac}\right)}^{+}+2{\;\mathrm{OH}}_{\left(\mathrm{ac}\right)}^{-}$$

$${\mathrm{Zn}}_{\left(\mathrm{ac}\right)}^{2+}+2{\;\mathrm{OH}}_{\left(\mathrm{ac}\right)}^{-}\overset{}{\to }\;\mathrm{Zn}{\left(\mathrm{OH}\right)}_{2\;\left(s\right)}$$

$$\mathrm{Zn}{\left(\mathrm{OH}\right)}_{2\;\left(s\right)}\;\overset{\Delta }{\to }{\;\mathrm{ZnO}}_{\left(s\right)}+H_{2}O_{\left(g\right)}$$

Mechanism 2

$$\mathrm{Zn}{\left({\mathrm{CH}}_{3}\mathrm{COO}\right)}_{2}\times 2{\;H}_{2}O_{\left(\mathrm{aq}\right)}+2{\;\mathrm{TEA}}_{\left(\mathrm{aq}\right)}\overset{}{\to }\;\mathrm{Zn}{\left[\mathrm{TEA}\right]}_{\left(\mathrm{aq}\right)}+2{\;\mathrm{CH}}_{3}{\mathrm{COO}}_{\left(\mathrm{ac}\right)}^{-}$$

$$\mathrm{Zn}{\left[\mathrm{TEA}\right]}_{\left(\mathrm{aq}\right)}\overset{}{\to }{\;\mathrm{Zn}}_{\left(\mathrm{ac}\right)}^{2+}+{\mathrm{TEA}}_{\left(\mathrm{aq}\right)}$$

$$2{\;\mathrm{NaOH}}_{\left(s\right)}\overset{{\;H}_{2}O\;}{\to }2{\;\mathrm{Na}}_{\left(\mathrm{ac}\right)}^{+}+2{\;\mathrm{OH}}_{\left(\mathrm{ac}\right)}^{-}$$

$${\mathrm{Zn}}_{\left(\mathrm{ac}\right)}^{2+}+2{\;\mathrm{OH}}_{\left(\mathrm{ac}\right)}^{-}\overset{}{\to }\;\mathrm{Zn}{\left(\mathrm{OH}\right)}_{2\;\left(s\right)}$$

$$\mathrm{Zn}{\left(\mathrm{OH}\right)}_{2\;\left(s\right)}+2{\;\mathrm{OH}}_{\left(\mathrm{ac}\right)}^{-}\overset{}{↔}\;\mathrm{Zn}{\left(\mathrm{OH}\right)}_{4\;\left(\mathrm{aq}\right)}^{2-}$$

$$\mathrm{Zn}{\left(\mathrm{OH}\right)}_{4\;\left(\mathrm{aq}\right)}^{2-}\;\overset{}{\to }{\;\mathrm{ZnO}}_{\left(s\right)}+H_{2}O_{\left(g\right)}+2{\;\mathrm{OH}}_{\left(\mathrm{ac}\right)}^{-}$$

Even though both mechanisms are quite similar, the addition of TEA plays an outstanding role in the morphology of ZnO NPs enhancing the conversion from Zn(OH)_2_ to ZnO.

## 5. Bioorganic Material in Sol-Gel Preparation

The Pechini synthesis ensures that metal ions are combined and stabilized homogeneously within a covalent matrix or ‘gel’ by synthesizing a polymer in situ. Consequently, it seems natural that new variants consider the direct synthesis of Sol-Gel precursors from natural or synthetic polymers. The polymers create well-defined structures in solution, and metal ions have strong interactions with them. The ability of some polymers to create ordered superstructures can be used to control the morphology of the desired material.

Authors such as Liu et al. [94], Shao et al. [95] and Yong-Jin Han et al. [96] have worked with organic compounds and zinc or vanadium oxide [97,98] obtain new functional materials such as supercapacitors [99,100], taking advantage of the benefits offered by organic materials in relation to the natural and structured arrangement provided by the different natural sources from which they are obtained.

Yoseph Bar-Cohen coined the statement that “…through evolution, nature has ‘experimented’ with various solutions to its challenges and has improved the successful ones” [101]. Materials as versatile as activated carbon [102,103,104], porous carbon [105], graphene [106,107] and carbon nanotubes (CNT) [108,109,110] have been considered as a prospective type of electrode materials for supercapacitors due to their economic acquisition, high surface area, excellent electrical conductivity and outstanding electrochemical stability [111,112]. Each of these materials, however, has drawbacks. Commercial activated carbon, for example, has a poor specific capacitance due to its small surface area [113]. On the other hand, CNTs and graphene have a large specific surface area and excellent electrical conductivity, but their manufacturing process is complex and of high economic cost [114]. Furthermore, graphene spontaneously agglomerates and stacks during the production process, limiting its synthesis and large-scale application [115,116].

**Figure 8:**
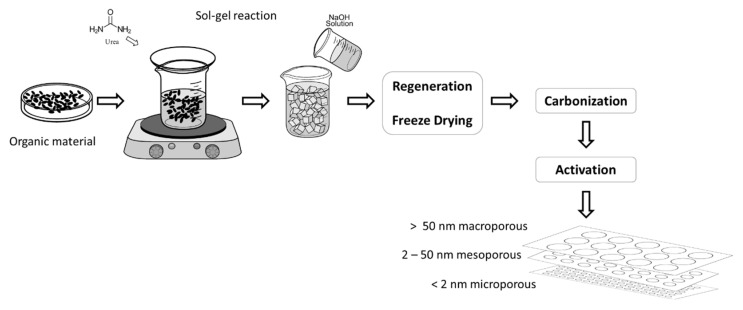
Schematic representation of the fabrication of highly porous bagasse-derived carbon aerogels.

Therefore, most studies have focused on obtaining porous carbon materials due to their versatility at the time of fabrication and the physicochemical advantages over other types of carbon-based materials. Authors such as Hao et al. obtained hierarchical porous carbon (HPC) aerogel from sugarcane waste products containing mainly cellulose derivatives, bagasse. The porous material was obtained by carbonization of the freeze-dried bagasse aerogel [117] (see Figure 8). The authors’ preparation method, by Sol-Gel reaction, results in carbon aerogels with hierarchical macro-, meso- and microporous structures and possessing a high specific surface area 1892.4 m^2^ g^−1^, whose specific capacity was 142 F g^−1^ at 0.5 A g^−1^.

**Figure 9:**
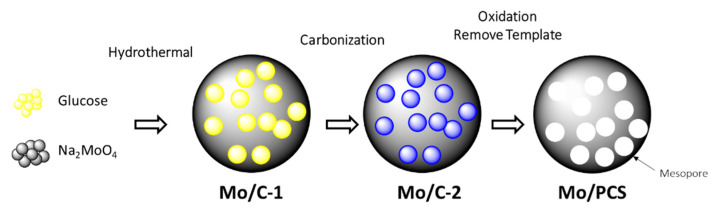
Schematic representation of the fabrication of the porous carbon spheres (PCS).

A lot of effort has gone towards designing porous carbon spheres (PCSs) using hydrothermal carbonization [118,119]. Liu et al., for example, used Pluronic F127 as a soft template to generate carbon spheres with organized mesopores and a large surface area (1100 m^2^ g^−1^) during hydrothermal treatment of D-fructose. In a three-electrode configuration, the produced spheres had a high capacitance of 350 F g^−1^ at 1.0 A g^−1^, and a capacitance of 190 F g^−1^ at 2.0 A g^−1^ in a two-electrode configuration [120]. A year later, Wang’s group used hydrothermal treatment of glucose and sodium molybdate (Na_2_MoO_4_ as a porogen agent to create porous carbon spheres) to create PCSs with a specific capacitance of 260 F g^−1^ outstanding high-speed performance, and strong long-term cycle stability. Alginic acid is carbonized hydrothermally and then activated with KOH [121]. A brief summary of porous carbon spheres is shown in Figure 9.

Hydrothermal carbonization is a strong, cost-effective, and ecologically acceptable method for converting organic molecules into usable carbon materials. Under mild hydrothermal conditions, a variety of organic compounds such as glucose, sucrose, starch, fructose, alginic acid, among others, can be transformed into carbonaceous spheres [122].

Pan et al. obtained a porous carbon from asphalt, whose properties highlight its high specific capacity of 277 F g^−1^ at a current density of 0.05 A g^−1^. According to the authors, the ultrathin and porous structure reduces resistance by minimizing the diffusion distance of electrolyte ions and aids the plentiful porosity channels as active locations for ion transport and storage [123].

These precedents utilized organic wastes to make porous carbon with good capacitance performance, opening a novel way to transform organic wastes into supercapacitor material and lowering carbon material costs. The specific capacitance of these carbon materials, on the other hand, has yet to increase their scalability. Therefore, it is important to prepare carbon materials with high specific capacity from low-cost biomass feedstock.

These precedents utilized organic wastes to make porous carbon with good capacitance performance, opening a novel way to transform organic wastes into supercapacitor material and lowering carbon material costs. The specific capacitance of these carbon materials, on the other hand, has yet to increase their scalability. Therefore, it is important to prepare carbon materials with high specific capacity from low-cost biomass feedstocks.

In this sense, Kong et al. employed maize starch as a carbon precursor, which they synthesized by dissolving corn starch in KOH solution and fabricating a starch-based gel after the water was removed. Finally, 3D-reticular porous carbon (3D-RPC) was created by carbonizing a starch gel and activating KOH in situ (a process like those described by Hao et al.). The advantages of 3D-RPC include a wide surface area, high porosity and an optimum pore size distribution. The macropores formed with this new material (3D-RPC) are primarily from the intermediate space between the three-dimensional starch gel networks, whereas the KOH activation process produced mesopores and micropores. The material’s porosity displayed exceptional supercapacitive performance with a 372 F g^−1^ specific capacitance (0.5 A g^−1^). The electrochemical performance of 3D-RPC outperforms that of most previously characterized carbon materials, opening a new path for large-scale production of a low-cost, high-purity porous carbon for supercapacitors [124].

## 6. Conclusions

Sol-Gel method offers a unique synthesis route to produce novel and tailor-made nanomaterials with full control of morphology, size, composition, crystallinity, porosity enhanced by combustion or hydrothermal/solvothermal treatment. Nevertheless, some precautions must be taken in order to obtain the best results. The nanoparticle composition must be analyzed before running the synthesis (pH, working temperature, reaction time, metal cation precursors mixtures). The morphology and size must be also taken into account, e.g., organic surfactants or molecules are required throughout the process. The velocity of both hydrolysis and condensations reactions should be investigated extensively, this is the key stage that leads into the final product and also confers the distinctive feature in every synthesis.

Sol-Gel also offers the possibility to produce in situ doped nanomaterial and mixed lattices. However, a precise control of doping requires full-knowledge of the different equilibriums that take place during hydrolysis and condensation reactions. When doping cation precursors are added, soft chemistry must also be used to avoid fast alterations from the final product.

The size distribution and aspect ratio might be another challenging task to improve. Although the use of organic template molecules as structure directors in many syntheses generates great results, most of the time, secondary phases are originated that affect the performance in different applications.

Sol-Gel processes in the future could be assisted with photocatalysts during synthesis. This could induce the formation of desirable doping processes or could reduce the formation of secondary phases. The use of biomaterials in the synthesis of Sol-Gel, especially organic waste, offers a novel low-cost and ecological platform for the manufacture of functional materials.

## Figures and Tables

**Table 1:** Main characteristic of methods for synthesis by Sol-Gel.

Synthesis Methods	Particle Size	Agglomeration	Purity	Precursors	Calcination Temperature	Observations
Sol-Gel	>10 nm	Moderate	Excellent	Alkoxide or Acetylacetonates	800 °C	Obtaining of uniform and small sized powders
Sol-Gel Pechini	>10 nm	Moderate	Excellent	Nitrates	800–1000 °C	Accurate control of the final material composition

## Data Availability

Not applicable.
